# Three-dimensional printing and computer navigation assisted hemipelvectomy for en bloc resection of osteochondroma: A case report

**DOI:** 10.1097/MD.0000000000006414

**Published:** 2017-03-24

**Authors:** Yaqing Zhang, Lianjiang Wen, Jun Zhang, Guoliang Yan, Yue Zhou, Bo Huang

**Affiliations:** Department of Orthopedics, Xinqiao Hospital, The Third Military Medical University, Chongqing, China.

**Keywords:** computer navigation, hemipelvectomy, en bloc resection, osteochondroma, three-dimensional printing

## Abstract

**Rationale:**

Three-dimensional (3D) printed templates can be designed to match an individual's anatomy, allowing surgeons to refine preoperative planning. In addition, the use of computer navigation (NAV) is gaining popularity to improve surgical accuracy in the resection of pelvic tumors. However, its use in combination with 3D printing to assist complex pelvic tumor resection has not been reported.

**Patient concerns:**

A 36-year-old man presented with left-sided pelvic pain and a fast-growing mass. He also complained of a 3-month history of radiating pain and numbness in the lower left extremity.

**Diagnoses:**

A biopsy revealed an osteochondroma with malignant potential. This osteochondroma arises from the ilium and involves the sacrum and lower lumbar vertebrae.

**Interventions:**

Here, we describe a novel combined application of 3D printing and intraoperative NAV systems to guide hemipelvectomy for en-bloc resection of the osteochondroma. The 3D printed template is analyzed during surgical planning and guides the initial intraoperative bone work to improve surgical accuracy and efficiency, while a computer NAV system provides real-time imaging during the tumor removal to achieve adequate resection margins and minimize the likelihood of injury to adjacent critical structures.

**Outcomes:**

The tumor mass and the invaded spinal structures were removed en bloc.

**Lessons:**

The combined application of 3D printing and computer NAV may be useful for tumor targeting and safe osteotomies in pelvic tumor surgery.

## 1 Introduction

The common clinical symptoms of osteochondroma with malignant potential are pain and mass enlargement.^[1,2]^ Resection of the tumor is suggested if the tumor increases in size and progresses toward malignancy. During surgical resection, the entire lesion should be removed to minimize the chance of reoccurrence.^[1]^ Therefore, en-bloc resection is the treatment of choice for such lesions when feasible. However, the en-bloc resection of pelvic tumors is challenging due to the complex three-dimensional (3D) geometry of the pelvic bone, limited visibility, and the proximity to delicate organs and structures such as the bladder, sciatic nerve, and numerous vessels.

In recent years, the use of computer navigation (NAV) has been described in the surgical resection of musculoskeletal tumors such as those of the pelvis and sacrum.^[3,4]^ Such systems have been developed to improve surgical accuracy with the goal of achieving adequate resection margins and better oncologic results.^[5]^ However, the virtual image seen on the NAV console is based entirely on bone anatomy. After surgical manipulation of the bone anatomy, the real-time anatomic location may not be accurately reflected.

Three-dimensional (3D) printing is also emerging as a clinically promising technology for customizing anatomic models and allowing surgeons to examine patient anatomy in a more concrete way than traditional radiological images.^[6]^ The model is able to accurately display tumor features, and the preoperative analysis of 3D printed models has resulted in surgeons reporting major improvements in their understanding of anatomic and surgical complexity.^[7]^

We combined 3D printing and a computer NAV system for preoperative planning and intraoperative real-time localization of a complex pelvic tumor and were able to perform en bloc resection with adequate surgical margins.

## 2 Case report

**Figure 1:**
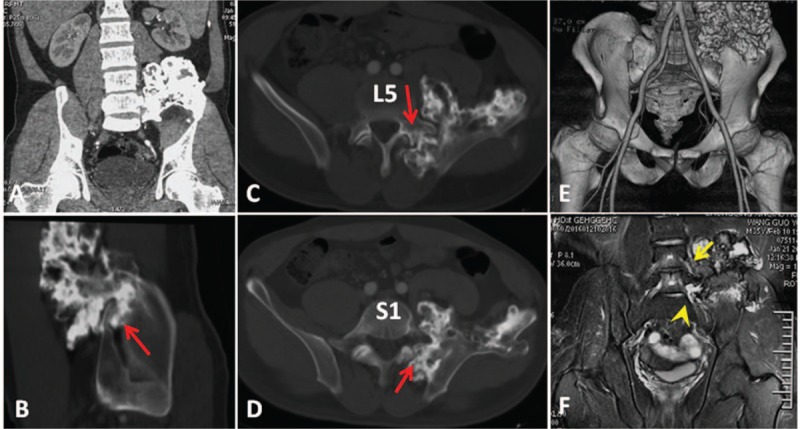
(A) Preoperative coronal, (B) sagittal, and (C, D) axial CT scans showing that the tumor (red arrow) involves the ilium, left sacroiliac joint, left transverse process, pedicle, and vertebral body of L5 and the sacrum. (E) The anterior aspect of the preoperative 3-dimensional computed tomography scan displays the proximity of the iliac vessels and the tumor. (F) Magnetic resonance image demonstrating that the tumor is adhesive to the L4 (yellow arrow) and L5 (yellow arrow head) nerve roots.

A 36-year-old man presented with left-sided pelvic pain and a fast-growing mass. The pelvic pain had slowly worsened over 2 months. He also complained of a 3-month history of radiating pain and numbness in the lower left extremity. Magnetic resonance imaging (MRI), computed tomography (CT), and a 3D printed model revealed an iliac tumor invading the sacrum, left sacroiliac joint, left facet joint of L5/S1, left transverse process, pedicle, and partial L5 vertebral body (Fig. 1). The CT scan was strongly suggestive of pelvic osteochondroma. The MRI also revealed that the tumor may have been adhesive to the L4 and L5 nerve roots, which may have been responsible for the pain radiating down the leg (Fig. 1). A biopsy revealed an osteochondroma with a grossly irregular surface, actively proliferating chondrocytes, increased thickness of the cartilage cap, and irregular mineralization.

**Figure 2:**
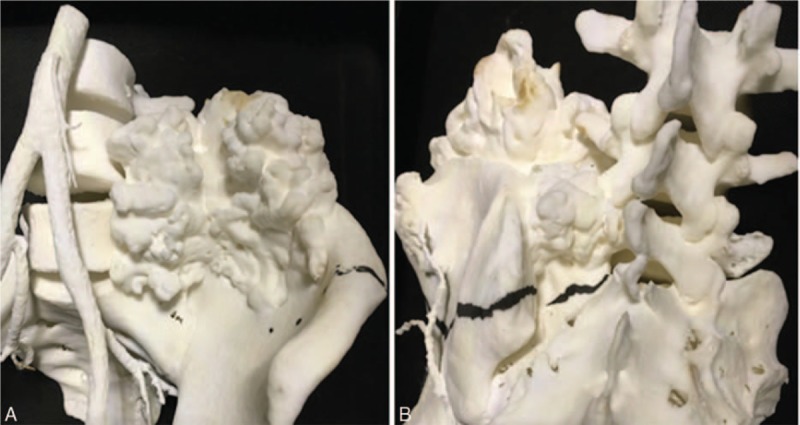
Photographs showing the (A) anterior and (B) posterior aspects of the 3-dimensional printed model.

We planned to perform en bloc resection of the pelvic osteochondroma. Three-dimensional printing was employed for the preoperative planning (Fig. 2). The patient's pelvic CT scans were processed using Blender, an open-source image processing software. The resulting files were uploaded and printed by a commercial company (Natong Technology Co., Ltd., Beijing, China). The 3D printing processing chain includes CT scanning, blender processing, mesh processing, and 3D printing. The model printed with thermoplastics could be autoclave-sterilized and used for intraoperative guidance.

During the surgery, the patient was placed on a radiolucent table in a prone position under general anesthesia. Preoperative antibiotics were administered before any incisions. Intraoperative somatosensory-evoked potentials were used throughout the operation to monitor the patient's neurologic function.

### 2.1 Operative step 1: Normal bone removal using 3D model guidance to expose the tumor

**Figure 3:**
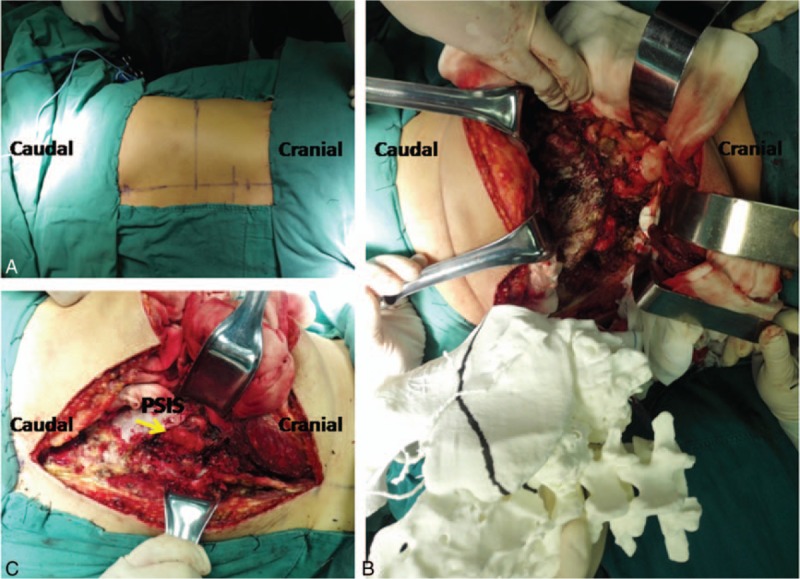
(A) The incision was converted to a T shape from L4/L5 to the left side. (B, C) Intraoperative photograph showing the use of 3-dimensional model guidance for normal bone removal to expose the tumor (yellow arrow). PSIS = posterior superior iliac spine.

A posterior approach was used and a midline incision extending from the spinous process of L3 to S3 was made. The incision was converted to a T shape from L4/L5 to the left side, exposing the posterior iliac crest completely (Fig. 3). The sterilized 3D printed model provided intraoperative guidance during surgical manipulation of the normal bone anatomy to enable appropriate exposure for the tumor resection (Fig. 3). Using true-size model guidance, resection of the spinal process and the right semi-lamina of L5 was performed to expose the osteotomy margin of the L5 vertebral body, while the invaded portion (left semi-lamina, transverse process, pedicle, and partial vertebral body) of L5 was left with the tumor resected. After complete S1 laminectomy, the osteotomy margin on S1 vertebral body was also exposed for the following en bloc resection of the tumor.

### 2.2 Operative step 2: Navigated tumor resection

**Figure 4:**
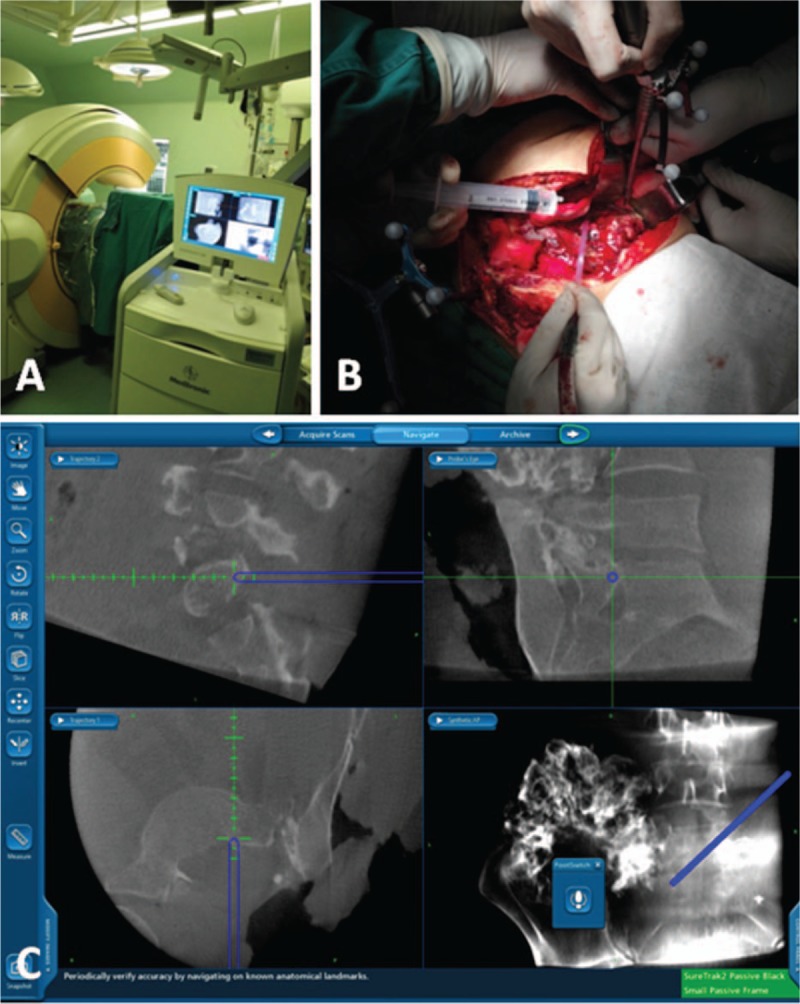
(A) Intraoperative photograph showing the use of the O-arm computer navigation system during the en bloc tumor resection. (B) Intraoperative photograph showing the reference array clamped to the right ilium and a tracking probe attached to the drill. (C) Views (coronal, sagittal, cross-sectional, and 3-dimensional) of the navigational system computer workstation.

Following the initial bone work, a reference array was clamped to the right ilium. An intraoperative CT scan was performed by a cone-beam CT (O-arm; Medtronic, Minneapolis, MN) and the CT data sets were automatically transferred to the stereotactic NAV system (StealthStation; Medtronic, Minneapolis, MN). A drill with a tracking probe was then registered to enable the precise trajectory of the drill to be tracked in real time in the axial and sagittal planes during the tumor removal (Fig. 4). Using NAV guidance, we identified the osteotomy margin on the left ilium, which was a minimum of 10 mm from the tumor margin. The locations of the planned osteotomies were marked with drill holes on the bone surface of the ilium, and the holes were connected using an osteotome to improve efficiency. On the spinal side, a vertical sagittal osteotomy was performed just medial to the left pedicle of L5 and a transverse osteotomy was adjoined to the superior border of the left foramina of S1 using the drill. Image-guided drill movements were tracked in real time to prevent an incorrect osteotomy direction. Partial resection of the sacroiliac joint was then performed by the navigated drill. Once the osteotomies were completed, the specimen became movable. When the specimen was mobilized and rotated laterally, the L4 and L5 nerve roots were gently dissected away from the tumor. After detachment of the soft tissue, the tumor mass and the invaded spinal structures were removed en bloc from the operative field.

### 2.3 Operative step 3: Reconstruction

**Figure 5:**
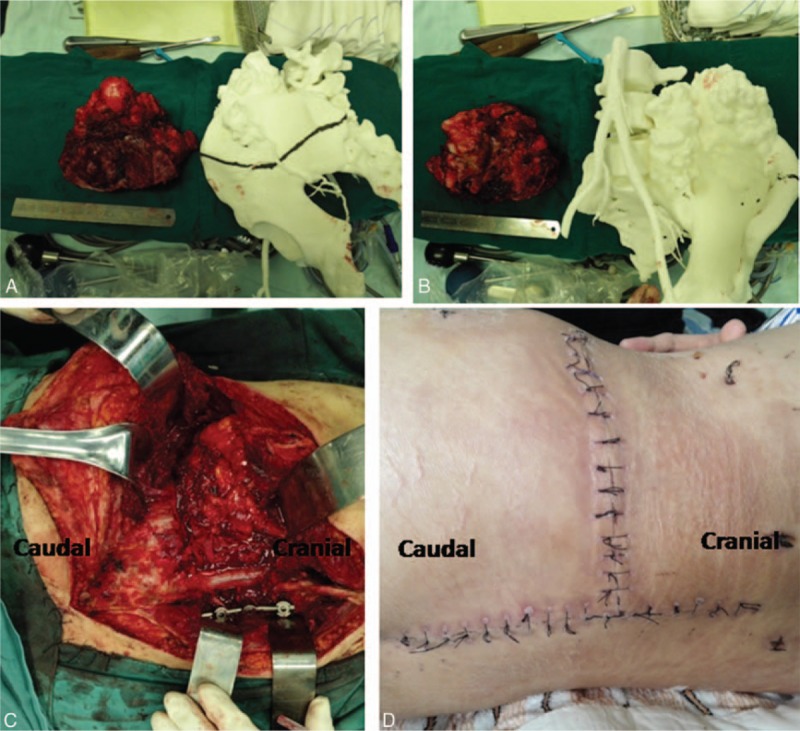
(A, B) Photograph of the specimen with adequate surgical margins obtained by en bloc resection. (C) Intraoperative photograph showing transforaminal lumbar interbody fusion as well as pedicle screw instrumentation placed from L4 to S1 on the right side. (D) Postoperative photograph showing the wound closure of the posterior-only approach.

**Figure 6:**
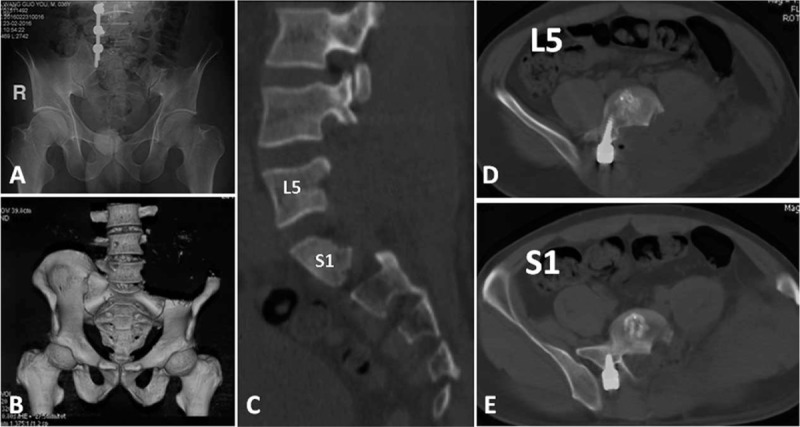
(A) Postoperative anteroposterior radiograph, (B) 3-dimensional computed tomography scan, and (C) sagittal and (D, E) axial images showing the bone resection extent and instrumentation position.

Transforaminal lumbar interbody fusions (TLIFs) as well as pedicle screw instrumentation were placed from L4 to S1 on the right side to provide lumbosacral arthrodesis (Figs. 5 and 6). No additional lumbopelvic reconstruction was required on the left side because of minimal resection of the sacroiliac joint (Fig. 6). The operative time was 380 minutes.

Histological analysis of the surgical specimen confirmed the diagnosis of osteochondroma with malignant potential, and the margins were negative for tumor in the final evaluation. The patient was neurologically intact. Although his postoperative course was complicated by gangliitis in the lower left extremity 1 week after the operation, symptom relief was achieved with a 2-week treatment course of pregabalin (75 mg p.o. b.i.d.).

Signed written consent was obtained from the patient for the publication of this case report.

## 3 Discussion

Here, we describe the combined use of 3D printing and a computer NAV system to enable the en bloc resection of a complex pelvic osteochondroma progressing toward malignancy. In this case, the tumor involved the ilium, sacrum, and left sacroiliac joint as well as the lumbar vertebrae. For the preoperative planning, the 3D printed model was used to accurately display the tumor features. The model provided an accurate depiction of the tumor and the adjacent critical structures to enable more direct and accurate preoperative planning. Intraoperatively, the 3D printed model also provided guidance for the surgical manipulation of the normal bone anatomy, while the intraoperative stereotactic NAV was used only for tumor targeting after the initial bone work. The initial bone removal is crucial to hemipelvectomy success. The bone work must be wide enough to allow preservation of the nerve roots and appropriate exposure for the subsequent tumor resection.^[8]^ To improve accuracy, the 3D model was autoclave-sterilized and used for intraoperative guidance during the surgical manipulation. Thereafter, we employed the O-arm coupled with stereotactic NAV to determine adequate tumor margins using intraoperative real-time localization. Experience has shown us that the virtual image seen on the NAV console may not be accurate after surgical manipulation of soft tissues and bone anatomy; therefore, we avoided using the NAV for the initial bone work.

Computer NAV-assisted surgery has been a useful method to increase accuracy in tumor resection of the pelvis since 2004.^[4,5,9]^ The use of a multi-dimensional anatomy imaging system improves surgeons’ visualization over conventional 2-dimensional imaging systems. However, previous CT-based computer NAV systems required patients to undergo another preoperative CT scan. In addition, CT images are acquired in the supine position, whereas spinal surgery is normally performed in the prone position. Thus, there is concern about NAV inaccuracies regarding movement at the intervertebral level in the mobile spine.^[10]^ The combination use of the O-arm and the StealthStation NAV offers a seamless NAV solution for spinal surgeries. This solution used with an intraoperative CT scan provides a 26-second scan time and automatic data transfer, eliminating the need for lengthy patient registration, reducing X-ray exposure for surgeons, and enabling NAV immediately after image acquisition. Because the CT images are acquired intraoperatively by the O-arm, they reveal the most current information about tumor extent. Furthermore, we found that the process was more effective when we checked real-time NAV images against the 3D printing model during the operation (380 minutes). Thus, this case demonstrates an effective en bloc tumor resection technique. However, this system's drawback is that the O-arm requires a radiolucent operative table for CT scanning, which incurs extra cost. Another unsolved problem is that the NAV system cannot simultaneously navigate both the soft tissues and the bone, while most tumors invade the surrounding soft tissue as well.

The use of image-guided drills for tumor resection in computer-assisted spine surgery was recently reported.^[11]^ In this case, a drill with high-speed bur was registered and tracked in real time with reference to the patient's anatomy, making it possible to check every bur movement moment-by-moment during the osteochondroma removal. The drill holes were also marked on the bone surface for the planned osteotomy locations. When the osteotomy accessed the structures anterior to the sacrum and ilium, a diamond bur was supplemented to avoid injuring important vessels and organs using NAV and 3D model guidance. For pelvic tumor excisions, the use of an image-guided drill is more stable and convenient than that of an osteotome.

In this case, we used the T-shape posterior-only approach to hemipelvectomy. The approach has been reported by the Mayo Clinic and Johns Hopkins University School of Medicine.^[12]^ The approach has advantages over traditional procedures: surgical morbidity is reduced by avoiding the anterior approach; the horizontal incision provides additional lateral access for tumor resection; and appropriate lumbopelvic reconstruction can be performed simultaneously. Moreover, wound closure is important for pelvic tumors. Complex closures may also require skin grafting or pedicled flap reconstruction, while the posterior-only approach may be simpler and shows a lower wound infection rate.^[12]^ The wound healed uneventfully in our patient 2 weeks after the operation (Fig. 5). However, 1 drawback of the posterior-only approach is the decreased access to the structures anterior to the sacrum and ilium. Therefore, use of the personalized 3D model here helps us to understand the normal and abnormal regional anatomy anterior to the pelvis.

The current case is the first reportofthe combined use of 3D printing and computer NAV for preoperative planning and intraoperative guidanceof vertebral osteotomies. The use of these useful tools ensures precise and safe osteotomies in pelvic tumor surgery via a posterior approach.
